# Improved Hydrogen Generation of Al-H_2_O Reaction by BiOX (X = Halogen) and Influence Rule

**DOI:** 10.3390/ma15228199

**Published:** 2022-11-18

**Authors:** Lumin Liao, Jiaxi Liu, Tao Wang, Fen Xu, Lixian Sun, Tianhao Zhou, Jinfan Wu, Yanxun Guan, Yumei Luo, Yongjin Zou, Hailiang Chu

**Affiliations:** 1 Guangxi Key Laboratory of Information Materials & Guangxi Collaborative Innovation Center for Structure and Properties for New Energy and Materials, School of Material Science and Engineering, Guilin University of Electronic Technology, Guilin 541004, China; 2 School of Electronic Engineering and Automation, Guilin University of Electronic Technology, Guilin 541004, China

**Keywords:** hydrogen generation, Al-H_2_O reaction, BiOX (X = F, Br and I), mechanism, density functional theory

## Abstract

In this work, three additives BiOX (BiOI, BiOBr, and BiOF) for Al-H_2_O reaction have been synthesized using chemical methods. SEM analysis shows that the structure of BiOF is nanoparticles, while BiOBr and BiOI have flower-like structures composed of nanosheets. Then, Al-BiOI, Al-BiOBr, and Al-BiOF composites have been prepared using the ball milling method. The effect of halogen ions on the performance of hydrogen generation from Al hydrolysis has been explored. The results indicate that the conversion yields of Al-BiOBr, Al-BiOI, and Al-BiOF for hydrogen generation are 96.3%, 95.3%, and 8.9%, respectively. In particular, the maximum hydrogen generation rate (MHGR) of Al-BiOI is as high as 3451.8 mL g^−1^ min^−1^, eight times higher than that of Al-BiOBr. Furthermore, the influence rule of BiOX (X = F, Cl, Br, and I) on Al-H_2_O reaction has been studied using density functional theory. The results illustrate that HI can be more easily adsorbed on the Al surface as compared with HF, HCl, and HBr. Meanwhile, the bond length between halogen ions and the Al atom increased in the order of F^−^, Cl^−^, Br^−^, and I^−^. Therefore, the dissociation of I^−^ from the Al surface becomes easier and will expose more active sites to enhance the reaction activity of Al. In summary, the BiOI has the most favorable performance to Al-H_2_O reaction.

## 1. Introduction

Hydrogen is a clean, efficient, safe and sustainable secondary energy source [1,2,3]. In the context of the global energy revolution and transformation, the strategic significance of developing hydrogen energy has become progressively noticeable [4]. Nonetheless, the large-scale utilization of hydrogen energy is inseparable from the efficient and convenient hydrogen generation technology [5,6]. The development of accessible, sufficient and economical hydrogen production methods is of great importance for the “hydrogen economy”.

Lately, numerous strategies for hydrogen production, including electrolytic water hydrogen production [7], photocatalytic hydrogen production [8], biological hydrogen production [9], borane ammonia complex hydrolysis hydrogen production [10], metal hydrolysis hydrogen production [11,12], and so on, have gained popularity and advancement in the industry [7,8,9,10,11,12]. Among them, hydrogen production from Al-H_2_O reaction has drawn in incredible consideration from domestic and foreign researchers since aluminum possesses many benefits such as plentiful resources, minimal expense, light mass density and no pollution [13,14]. The fundamental issue of Al-H_2_O reaction for hydrogen production is that a dense oxide film with a thickness of roughly 50 Å forms on the aluminum surface in air and obstructs Al-H_2_O reaction. Al(OH)_3_ is also generated on the aluminum surface during Al-H_2_O reaction, which likewise hinders hydrogen production. Therefore, the removal of oxide film on the aluminum surface is the key to improve hydrogen generation by Al-H_2_O reaction.

Effective methods for oxide film removal have been reported. A simple method is to utilize alkaline substances such as NaOH, KOH and Ca(OH)_2_ [15,16], but strong alkaline solutions are highly corrosive, demanding on the reaction vessel, and have potential safety hazards during use. The removal of oxide films by mechanical forces is highly desirable. Uehara et al. [17] observed that aluminum and aluminum alloys produced hydrogen continuously when they were cut or drilled in water, and hydrogen production stopped once cutting or drilling was stopped, implying that fresh aluminum surfaces reacted more readily with water to release hydrogen. Ball milling is a current research hotspot, and the addition of oxides [18], carbon materials [19] or inorganic salts [19] as abrasives and activators could effectively enhance the hydrogen generation performance of Al-based composites. Al alloys formed by adding low-melting metals could improve the reactivity of aluminum, and the commonly used low-melting metals include Ga, In, Sn, and Bi [20,21]. Fan et al. [22] found that metal Sn or Bi could promote Al-H_2_O reaction at room temperature. Additionally, Bi was a better activator than Sn. With the help of theoretical calculation, Xu et al. [23] evidenced that the Bi adsorbed on the Al (111) crystalline surface could reduce the adsorption energy of Al to OH groups, resulting in the aluminum with clean surface continually to react with water.

Al-BiOCl prepared by ball milling had a good performance of hydrogen generation in our prior study [24]. The results illustrated that the synergistic effect of Al fresh surface produced and Bi, Bi_2_O_3_ and AlCl_3_ generated during ball milling significantly improved the hydrogen production of the materials. For instance, the hydrogen conversion yield of Al-BiOCl reached 91.6%, but its hydrogen generation rate was relatively slow (MHGR was only 491.4 mL g^−1^ min^−1^). 

In this work, three additives (BiOI, BiOBr and BiOF) were synthesized by chemical method and respectively doped into Al powder by ball milling to prepare a series of Al-based composites. The results showed that the Al-BiOX (X = I and F) composites exhibited good hydrogen generation performance. Particularly, the MHGR of Al-BiOI was eight times higher than that of Al-BiOBr, and twice higher than that of Al-BiOCl [24]. Additionally, then, the influence rule of BiOX (X = F, Cl, Br and I) on Al-H_2_O reaction has been further researched by theoretical calculations.

## 2. Materials and Methods

### 2.1. Reagents and Apparatus

Aluminum powders (average diameter of 10 μm, 99%) were provided by Angang Group Aluminum Powder Co., Ltd. (Anshan, China). KBr (99.92%) and Bi(NO_3_)_3_·5H_2_O (99.999%) were purchased from Alfa Aesar Co., Ltd. (Tianjin, China). KI (99%), NaF (99%), CH_3_COOH (99.9%), and ethylene glycol (C_2_H_6_O_2_, 99.7%) were obtained from Xilong Chemical Co., Ltd. (Shantou, China). All above reagents were starting materials.

### 2.2. Additive Preparation

#### 2.2.1. Preparation of BiOF

Dissolving 5 mmol Bi(NO_3_)_3_·5H_2_O in 40 mL ethylene glycol, and dissolving 5 mmol NaF in 40 mL ethylene glycol. The above two solutions were mixed with constant vigorous stirring, while pouring 200 mL of deionized water. The obtained white precipitate was filtered and finally dried in a 353.15 K oven for 5 h and calcined in a muffle furnace at 573.15 K for 2 h to obtain BiOF.

#### 2.2.2. Preparation of BiOBr

KBr (4 mmol) was put into a beaker containing 100 mL of anhydrous ethanol, and stirred for 1 h after ultrasonic treatment. Then, 4 mmol Bi(NO_3_)_3_·5H_2_O was added and stirred vigorously for 3 h. The mixture was reacted using the hydrothermal method at 453.15 K for 15 h. After cooling, the filtrate was washed, and then the precipitate was placed in a 353.15 K oven for vacuum drying for 10 h to obtain BiOBr.

#### 2.2.3. Preparation of BiOI

1 mmol of Bi(NO_3_)_3_·5H_2_O was dissolved in 1 mL of CH_3_COOH, then mixed with 10 mL of 0.1 M KI solution and stirred vigorously; they reacted quickly. It could be found that its color gradually changed from yellow to red. Then, the resulting solution was transferred to a reactor, where it continued to react in a 413.15 K oven for 24 h. After cooling, the filtered product was washed, and then the precipitate was vacuum-dried in a 353.15 K oven for 12 h to obtain BiOI.

### 2.3. Preparation of Al-BiOX (X = F, Br and I) Composite

In a glove box filled with argon, Al powders, BiOF, BiOBr or BiOI were weighed and placed in a ball mill jar containing 15 steel balls of 10 mm. Al-BiOF, Al-BiOBr or Al-BiOI composites were prepared by mechanical ball milling using a planetary ball miller (PM400, Retsch, Shanghai, China) at a speed of 250 rpm. 

### 2.4. Hydrogen Measurement and Characterization

Our previously published research [25] outlined the experimental equipment used to evaluate hydrogen generation performance. The hydrogen production of the samples was tested by the drainage method.

The phase structures of samples were probed by X-ray powder diffraction (XRD, D8-Advance, Bruker Co., Ltd., Karlsruhe, Germany) equipped with Cu Kα radiation. The samples were qualitatively analyzed using an X-ray photoelectron spectrometer (XPS, ESCALAB 250, Thermo Electron Co., Ltd., Shanghai, China). The types and valence states of the elements on the surface of the material were obtained through XPS analysis. Scanning electron microscopy (SEM, JSM-5600LV, JEOL Co., Ltd., Tokyo, Japan) was employed to observe the micromorphology of samples, and the corresponding elemental mapping was performed by energy dispersive spectroscopy (EDS). The heat released by the reaction of the Al-BiOI with water was measured using a membrane mixed steel cell on a C80 Calvet calorimeter (SETARAM C80, Setaram Co., Ltd., Lyons, France). 

### 2.5. Computational Methods

Theoretical calculation was performed with the Vienna Ab initio simulation package (VASP) based on the density functional theory (DFT) methods [26,27]. The projector-augmented wave (PAW) method was used to describe the interaction of the core electrons and nucleus with the valence electrons [28]. Exchange–correlation effects were described by the generalized gradient approximation (GGA) with the Perdew–Burke–Ernzerhof (PBE) functional [29]. The DFT-D3 correction method of Grimme was used for van der Waals (vdW) interactions [30]. The energy cutoff was set to 450 eV, whereas the total energy and forces convergence thresholds were set to 1 × 10^−5^ eV and 0.02 eV/Å, respectively. A vacuum spacing of about 10 Å was added to eliminate the interaction of adjacent periodic layers. The Al (111) surface model was used for all DFT calculations and used a unit cell with a, b = 11.20 Å. The Brillouin zone was sampled using the Monkhorst–Pack method with a 5 × 5 × 1 and 7 × 7 × 1 k-point mesh for geometry optimization and energy calculations. To quantify the change in charge density, the Bader charge was used for the charge density analysis.

The adsorption energies ($E_{\mathrm{ads}}$) of HX (X = F, Cl, Br and I) on the Al (111) surface were calculated using Equation (1):

(1) $$\;E_{\mathrm{ads}}=E_{\mathrm{HX}/\mathrm{Al}\;\left(111\right)}-E_{\mathrm{Al}\;\left(111\right)}-E_{\mathrm{HX}}$$

where $E_{\mathrm{HX}/\mathrm{Al}\;\left(111\right)}$ is the energy of the adsorbed on the Al (111) surface, $E_{\mathrm{Al}\;\left(111\right)}$ is the energy of the Al (111) surface, and $E_{\mathrm{HX}}$ is the energy of the isolated HX.

## 3. Results and Discussion

### 3.1. Characterization of BiOF, BiOBr and BiOI

**Figure 1:**
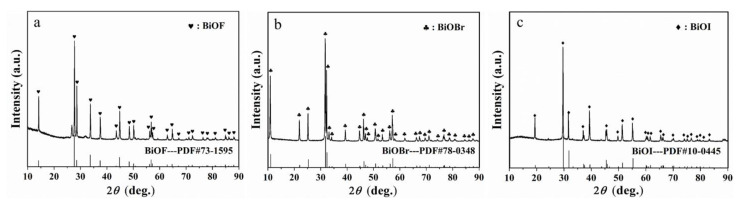
XRD patterns of (**a**) BiOF, (**b**) BiOBr and (**c**) BiOI.

As illustrated in Figure 1, the characteristic peaks of the three materials are consistent, respectively, with BiOF (standard PDF card #73-1595), BiOBr (#78-0348) and BiOI (#10-0445), illustrating that these dopants synthesized are BiOF, BiOBr and BiOI, respectively.

**Figure 2:**
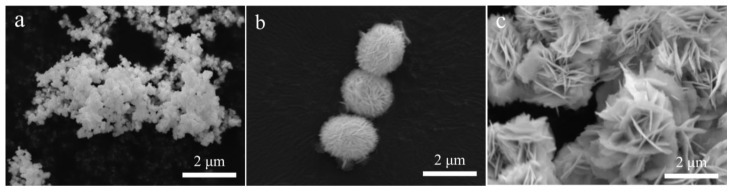
SEM images of (**a**) BiOF, (**b**) BiOBr and (**c**) BiOI.

To further investigate the microstructure of these additives, SEM analysis was performed (Figure 2). As shown in Figure 2a, the BiOF synthesized by the calcination method is nanoparticles, while both the BiOBr and BiOI synthesized by the hydrothermal method are in the form of stacked flower-like nanosheets (Figure 2b,c). Additionally, the order of the particle size of the three materials is BiOI > BiOBr > BiOF.

### 3.2. Hydrogen Generation Performances of Al-BiOX (X = F, Cl, Br and I)

The influence of the three dopants on the activity of Al powders was investigated and compared with that of Al-15 wt% BiOCl [24] (Table 1). Based on Table 1, Al-15 wt% BiOBr shows a remarkable conversion yield of 96.3%, which is the highest among the four materials, but its MHGR (430.9 mL g^−1^ min^−1^) is lower than that of Al-15 wt% BiOCl. The MHGR of Al-15 wt% BiOI is as high as 3451.8 mL g^−1^ min^−1^, which is seven times that of Al-15 wt% BiOCl and eight times that of Al-15 wt% BiOBr, and its conversion yield also reaches 95.3%. Accordingly, BiOI displays the best effect on the activation of Al among the four dopants. The possible reason is that I^−^ is the most reductive in the four halogen ions (F^−^, Cl^−^, Br^−^ and I^−^), and it is less stable and prone to lose electrons, thus accelerating the Al hydrolysis.

### 3.3. Effects of Doped Content of BiOI

Al-*x* wt% BiOI composites were prepared and evaluated for their hydrogen generation performance, as concluded in Table 2. From Table 2, both the conversion yield and MHGR first increase and then decrease with the increasing content of BiOI. When the BiOI content is 5 wt%, the conversion yield and MHGR are 54.6% and 704.9 mL g^−1^ min^−1^, respectively, while those of Al-15 wt% BiOI increased considerably, reaching 95.3% and 3451.8 mL g^−1^ min^−1^, respectively. Further increasing the BiOI content to 20%, the MHGR of the composite is 4545.9 mL g^−1^ min^−1^, but its conversion yield drops to 81.7%. As for Al-25 wt% BiOI, its conversion yield and MHGR are even lower because BiOI cannot produce hydrogen itself, and excessive doping of BiOI reduces its conversion yield.

### 3.4. Effects of Ball Milling Conditions

In addition to the composition of the initial sample, the ball milling conditions will also affect its property. In this part, the relevant preparation conditions were optimized.

A series of composites were prepared by adjusting the mass ratios of ball to powder. The hydrolysis properties of these composites are shown in Table 3. It can be concluded from Table 2 that the conversion yield first increases and then decreases, whereas most of them are above 90%. When the mass ratio of ball to powder is 60:1, the conversion yield reaches a maximum of 95.3%. Among these materials, the highest MHGR is 4217.6 mL g^−1^ min^−1^, which corresponds to the sample with a ball-to-powder ratio of 90:1. The MHGRs of the other four samples differs slightly, all within the range of 3000.0 mL g^−1^ min^−1^ to 4000.0 mL g^−1^ min^−1^. 

The hydrogen generation performances of Al-15 wt% BiOI were tested by changing ball milling time. The results are summarized in Table 4. The conversion yield and the MHGR of the composite correspond to 76.6% and 769.4 mL g^−1^ min^−1^ after 1 h of ball milling, while both are further enhanced to 87.5% and 2756.5 mL g^−1^ min^−1^ till they are ball-milled for 3 h. The best hydrolysis properties are achieved till 5 h ball milling. It has a hydrogen conversion yield of 95.3%, with a 1101.6 mL g^−1^ of H_2_ release and a MHGR of 3451.8 mL g^−1^ min^−1^ at room temperature. Extending the ball milling time to 7 h, the MHGR reaches 3688.2 mL g^−1^ min^−1^, but its conversion yield reduces to 93.9%.

**Figure 3:**
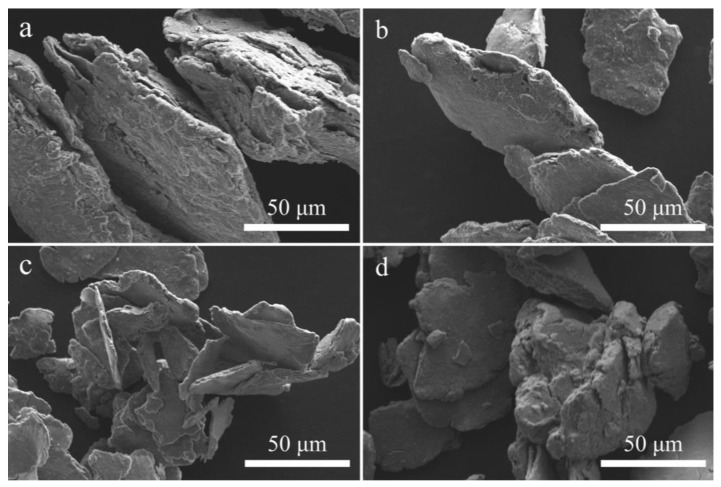
SEM images of Al-15 wt% BiOI at a ratio of ball to powder of 60:1 with ball milling for 1 h (**a**), 3 h (**b**), 5 h (**c**) and 7 h (**d**).

Figure 3 shows the SEM images of the above four samples. From Figure 3, it is evident that the shape of the Al-based composites after ball milling is lamellar accumulation. The layers separate from one other as the ball milling duration is extended to 5 h, and the particle size of the material is greatly reduced. When the ball milling time is increased to 7 h, cold welding agglomeration occurrs due to the high energy generated for prolonged ball milling, which seriously affects the hydrolysis properties of the material. This is why the hydrogen generation performance of the composite with 7 h ball milling was slightly lower. Combined with the above analysis, the ideal ball milling time is 5 h.

Table 5 lists the MHGR and hydrogen production of Al-based composites reported recently. Compared with the results of different materials, it seems the Al-BiOI sample exhibited relatively good hydrogen generation performance.

### 3.5. Effects of Reaction Temperature

**Figure 4:**
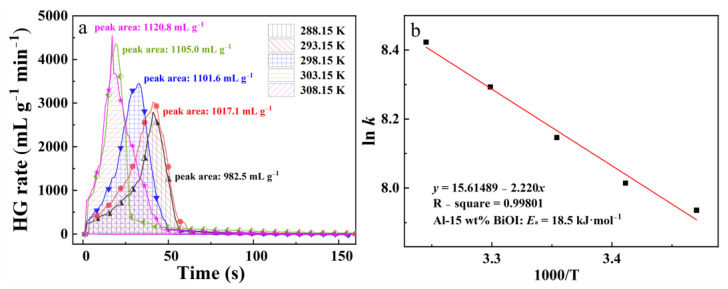
Curves of hydrogen generation rate (**a**) and a plot of ln *k* vs. 1000/T (**b**) of Al-BiOI.

Reaction temperature is a crucial factor affecting chemical reactions [36]. The Al-H_2_O reaction is exothermic; therefore, increasing the reaction temperature has a beneficial impact on the hydrogen generation performance of the Al-BiOI composite. The hydrolysis properties of Al-15 wt% BiOI were measured at different reaction temperatures. The results are shown in Figure 4a. As observed in Figure 4a, when the temperature is increased from 288.15 K to 308.15 K, the amount of hydrogen released from Al-15 wt% BiOI rises steadily from 982.5 mL g^−1^ to 1120.8 mL g^−1^, and the MHGR increases from 2940.0 mL g^−1^ min^−1^ to 4549.4 mL g^−1^ min^−1^. The results indicate that the hydrolysis kinetics of Al-BiOI are improved by raising the initial reaction temperature.

In light of the Arrhenius equation:

*k* = A·exp (−*E*_a_/R*T*) (2)

where *k* refers to the MHGR (mL g^−1^ min^−1^), A is the pre-exponential factor, *E*_a_ is the apparent activation energy, R is the gas constant (8.314 J mol^−1^ k^−1^), and *T* is the initial reaction temperature. Figure 4b provides a plot of ln *k* vs. 1000/T, and the apparent activation energy for the reaction of Al-15 wt% BiOI with water is 18.5 kJ mol^−1^. This value is far below that of the reaction between the Al-15 wt% BiOCl and water (26.9 kJ mol^−1^) [24], which further demonstrates that Al-15 wt% BiOI has a higher reactivity than that of Al-15 wt% BiOCl.

**Figure 5:**
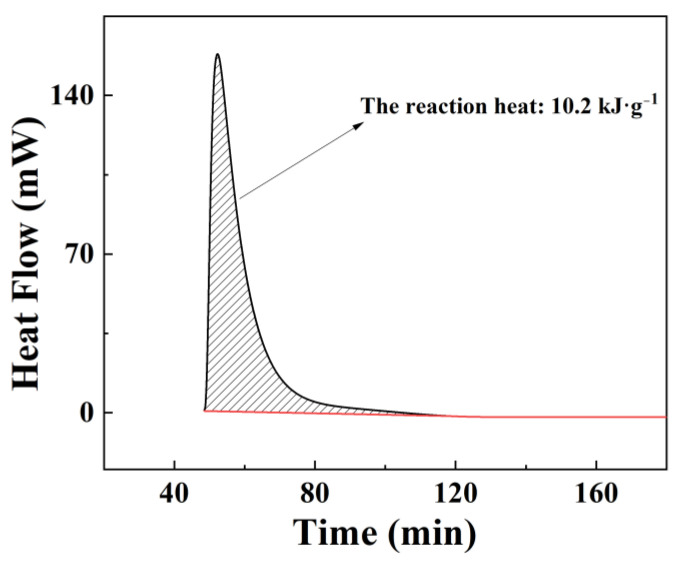
Reaction heat of Al-15 wt% BiOI with water.

**Figure 6:**
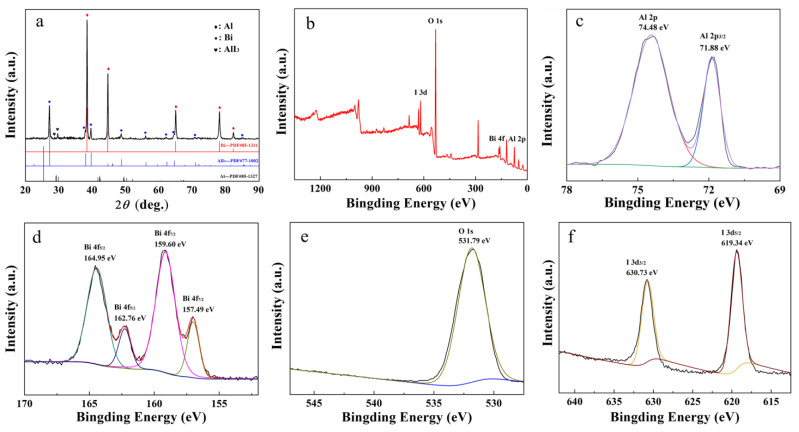
Characterization of Al-BiOI, (**a**) XRD; (**b**–**f**) XPS curves.

Meanwhile, a C80 calorimeter was used to measure the reaction heat of Al-15 wt% BiOI hydrolysis reaction. Figure 5 clearly shows that the reaction heat of Al-15 wt% BiOI with water is 10.2 kJ g^−1^. According to the heat of reaction between pure Al and water being 16.45 kJ g^−1^ [36], the theoretical reaction heat of Al-15 wt% BiOI should be 14.0 kJ g^−1^. The actual measured value is less than the theoretical value due to the solid-phase reaction between Al and BiOI during sample preparation, resulting in the consumption of a small amount of Al (see Figure 6a).

## 4. Mechanism Analysis

To reveal the hydrogen generation mechanism of Al-BiOI composite, XRD and XPS analysis were conducted on the composite material after mechanical ball milling, as indicated in Figure 6.

From Figure 6a, the BiOI peak in the Al-BiOI composite disappears after high-energy ball milling. At the same time, the characteristic peaks of elemental Bi and the faint characteristic peaks of AlI_3_ appear, demonstrating that a solid-phase reaction between BiOI and a small portion of Al occurrs in this process.

As shown in the XPS spectra of Figure 6b, Al-BiOI contains four elements (Al, Bi, O and I). In Figure 6c, the peaks of Al 2p at 74.48 eV and Al 2P_3/2_ at 71.88 eV are assigned to Al^3+^ and Al^0^ [37], respectively. In accordance with Figure 6d, the XPS spectra of Bi 4f_7/2_ and Bi 4f_5/2_ show four peaks. The peaks at 157.49 eV and 162.76 eV are associated with Bi^0^ [38], whereas the peaks at 159.60 eV and 164.95 eV are attributed to Bi^3+^ [38]. They illustrate Al-BiOI contains Bi and Bi_2_O_3_ after ball milling. As for Figure 6f, the peaks of I 3d_3/2_ at 630.73 eV and I 3d_5/2_ at 619.34 eV belong to I^−^ [39], indicating the formation of AlI_3_. 

According to the above results, the solid-phase reaction for Al and BiOI which occurs during ball milling can be described in Equation (3):

Al + 3BiOI → AlI_3_ + Bi + Bi_2_O_3_ (3)

**Figure 7:**
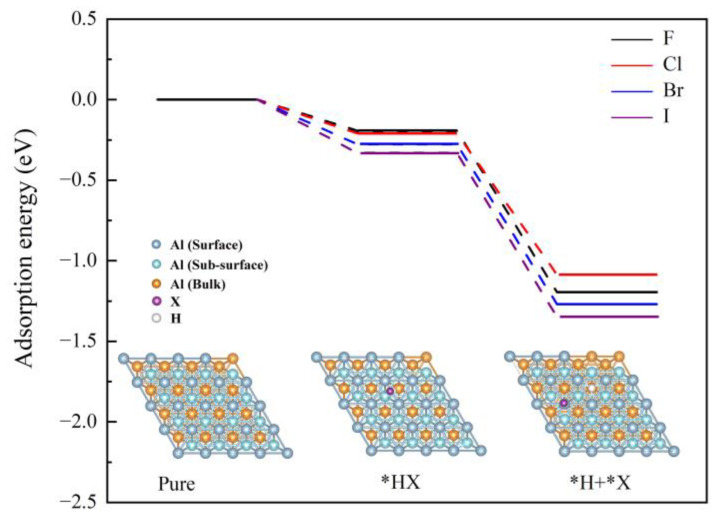
The adsorption energy potential profiles of HX cleavage on the Al (111) surface.

Based on the XRD and XPS analysis, it shows that the mechanism of action of BiOI is similar to that of BiOCl [24]. However, the hydrogen generation performance of Al-BiOI is much larger than that of Al-BiOCl. The reason is further analyzed through density function calculation (see Figure 7 and Table 6).

It appears that AlX (X = F, Cl, Br, and I) produced in situ during ball milling meets water to form HX. Generally, the radius of I is the largest among the above halogen ions, leading to the weakest binding capacity to H. Evidently, HI is more likely to dissociate to form H^+^, which enhances Al-H_2_O reaction. To further understand the influence rule of BiOX on Al hydrolysis for releasing hydrogen, a surface adsorption model *H + *X was constructed and the adsorption energy of the HX on the Al surface was evaluated using DFT calculation. The results show that the hydrogen production performance ranking is related to the adsorption energy of HX and the combining force of halogen ions on the Al (111) surface. As shown in Figure 7, HI had stronger adsorption energy on the Al surface, which induced more HI adsorbed on the Al (111) surface. Furthermore, the calculation results obtained by the model *H + *X are listed in Table 6. It can be found that the bond length between halogen ions and Al atom becomes longer in the order of F, Cl, Br and I, as well as the related charge transfer from the Al (111) surface to halogen ions being attenuated. Herein, the combining force of the halogen ions on the Al surface will be weakened and expose more active sites, which is beneficial to Al-H_2_O reaction.

## 5. Conclusions

In this paper, three additives (BiOF, BiOBr, and BiOI) were respectively prepared by chemical method. SEM analysis indicated that the structures of BiOBr and BiOI obtained were flowers stacked in nanosheets, while the structure of BiOF synthesized was nanoparticles. A series of Al-based hydrogen production materials (Al-BiOF, Al-BiOBr and Al-BiOI) were fabricated by ball milling. The results of the hydrogen generation performance tests showed that the activation ability of BiOX for Al-H_2_O reaction increased in the order of BiOF, BiOBr, to BiOI. The highest hydrogen production was achieved for Al-15 wt% BiOI ball-milled for 5 h (1101.6 mL g^−1^), and the MHGR was 3451.8 mL g^−1^ min^−1^. A low apparent activation energy of the reaction of Al-15 wt% BiOI with water was 18.5 kJ mol^−1^, and its reaction heat was 10.2 kJ g^−1^. The analysis of the hydrogen generation mechanism indicated that Al and BiOI underwent a solid-phase reaction during high-energy ball milling, forming AlI_3_, Bi, and Bi_2_O_3_ in situ. The synergistic effect of AlI_3_, Bi, Bi_2_O_3_, and the fresh Al surface produced by ball milling significantly enhanced the hydrogen generation performance of Al-BiOI. The results of DFT calculations showed that the HI adsorbed on the Al (111) surface had stronger adsorption energy to lead to more HI adsorption on the Al surface. Meanwhile, the results also demonstrated that the bond length between halogen ions and Al atom became longer in the order of F, Cl, Br, and I, and the charge transfer from the Al (111) surface to halogen ions was attenuated accordingly. Therefore, the dissociation process from the HI to H and I ions became easier, displaying that the I ion had the weakest binding on the Al surface and with more exposing active sites of Al to enhance the reaction activity of Al with water. This work is of guiding significance for the development of high-activity Al-based hydrogen production materials.

## Figures and Tables

**Table 1:** Hydrogen generation performances of Al-15 wt% BiOX (X = F, Cl, Br and I).

Samples	Amount of H_2_ mL g^−1^	Conversion Yield %	MHGR mL g^−1^ min^−1^
Al-15 wt% BiOF	103.1	8.9	666.0
Al-15 wt% BiOCl [24]	1058.1	91.6	491.4
Al-15 wt% BiOBr	1113.4	96.3	430.9
Al-15 wt% BiOI	1101.6	95.3	3451.8

**Table 2:** Hydrogen generation performances of Al-*x* wt% BiOI at 298.15 K.

Samples	Amount of H_2_ mL g^−1^	Conversion Yield %	MHGR mL g^−1^ min^−1^
Al-5 wt% BiOI	704.9	54.6	250.6
Al-10 wt% BiOI	988.1	81.5	571.8
Al-15 wt% BiOI	1101.6	95.3	3451.8
Al-20 wt% BiOI	888.8	81.7	4545.9
Al-25 wt% BiOI	759.0	74.4	2265.9

**Table 3:** Hydrogen generation performances of Al-15 wt% BiOI with different mass ratios of ball to powder at 298.15 K.

The Mass Ratio of Ball to Powder	Amount of H_2_ mL g^−1^	Conversion Yield %	MHGR mL g^−1^ min^−1^
30:1	1055.4	91.3	3261.2
45:1	1057.7	91.5	3314.1
60:1	1101.6	95.3	3451.8
90:1	1064.6	92.1	4217.6
120:1	1035.7	89.6	3974.1

**Table 4:** Hydrogen generation performances of Al-15 wt% BiOI with different ball milling times at 298.15 K.

Ball Milling Time h	Amount of H_2_ mL g^−1^	Conversion Yield %	MHGR mL g^−1^ min^−1^
1	886.5	76.6	769.4
3	1011.5	87.5	2756.5
5	1101.6	95.3	3451.8
7	1085.4	93.9	3688.2

**Table 5:** Comparison of Al-based composites.

Materials	Amount of H_2_ mL g^−1^	MHGR mL g^−1^ min^−1^	Reaction Temperature °C	Ref.
Al-BiOI	1101.6	3451.8	25	This work
Al-BiOCl	1058.1	491.4	25	[24]
Al-Bi-Zn	1223.0	390.4	20	[31]
Al-Bi(OH)_3_-NaCl	1020.0	2850.0	50	[32]
Al-Bi-Bi_2_O_3_	1170.0	1200.0	65	[33]
Al-nanoBi-GNS	925.0	978.0	20	[34]
Al-Ga-In-Sn-NiCl_2_	869.0	4060.0	25	[35]
Al-Ga-In-Sn-CoCl_2_	82.68	1634.0	25	[35]

**Table 6:** Calculated adsorption energies of *H + *X on Al (111) surface (E_ads_), the bond length between halogen and Al atoms (d), and the charge transfer from Al (111) to halogen ions.

Model	E_ads_ eV	d Å	Atomic Charge e
*H + *F	−1.195	1.707	0.850
*H + *Cl	−1.086	2.198	0.798
*H + *Br	−1.269	2.389	0.728
*H + *I	−1.348	2.647	0.655

* stands for an active site on the Al (111) surface.

## Data Availability

Not applicable.
